# Effect of Arginase Inhibition on Pulmonary L-Arginine Metabolism in Murine *Pseudomonas* Pneumonia

**DOI:** 10.1371/journal.pone.0090232

**Published:** 2014-03-03

**Authors:** Anne Mehl, Peyman Ghorbani, David Douda, Hailu Huang, Nades Palaniyar, Felix Ratjen, Hartmut Grasemann

**Affiliations:** 1 Program in Physiology and Experimental Medicine, SickKids Research Institute, The Hospital for Sick Children, and University of Toronto, Toronto, Ontario, Canada; 2 Division of Respiratory Medicine, Department of Pediatrics, The Hospital for Sick Children, and University of Toronto, Toronto, Ontario, Canada; 3 Department of Pediatric Pulmonology and Immunology, Charité - Universitätsmedizin Berlin, Berlin, Germany; 4 Pulmonary and Critical Care Medicine Division, Brigham and Women's Hospital and Harvard Medical School, Boston, Massachusetts, United States of America; National Institute of Agronomic Research, France

## Abstract

**Rationale:**

Infection of the lung with *Pseudomonas aeruginosa* results in upregulation of nitric oxide synthases (NOS) and arginase expression, and both enzymes compete for L-arginine as substrate. Nitric oxide (NO) production may be regulated by arginase as it controls L-arginine availability for NOS. We here studied the effect of systemic arginase inhibition on pulmonary L-arginine metabolism in *Pseudomonas* pneumonia in the mouse.

**Methods:**

Mice (C57BL/6, 8–10 weeks old, female) underwent direct tracheal instillation of *Pseudomonas* (PAO-1)-coated agar beads and were treated by repeated intra-peritoneal injections of the arginase inhibitor 2(S)-amino-6-boronohexanoic acid (ABH) or PBS until lungs were harvested on day 3 of the infection. L-arginine metabolites were quantified using liquid chromatography-tandem mass spectrometry, NO metabolites nitrate and nitrite by Griess reagent and cytokines by ELISA.

**Results:**

NO metabolite concentrations (48.5±2.9 vs. 10.9±2.3 µM, p<0.0001), as well as L-ornithine (29.6±1.7 vs 2.3±0.4 µM, p<0.0001), the product of arginase activity, were increased in *Pseudomonas* infected lungs compared to naïve controls. Concentrations of the NOS inhibitor asymmetric dimethylarginine (ADMA) were also increased (0.44±0.02 vs. 0.16±0.01 µM, p<0.0001). Arginase inhibition in the infected animals resulted in a significant decrease in L-ornithine (14.6±1.6 µM, p<0.0001) but increase in L-arginine concentration (p<0.001), L-arginine/ADMA ratio (p<0.001), L-arginine availability for NOS (p<0.001), and NO metabolite concentrations (67.3±5.7 µM, p<0.05). Arginase inhibitor treatment also resulted in an increase in NO metabolite levels in animals following intratracheal injection of LPS (p = 0.015). Arginase inhibition was not associated with an increase in inflammatory markers (IFN-γ, IL-1β, IL-6, MIP-2, KC or TNF-α) in lung. Concentrations of the L-ornithine-dependent polyamines putrescine, spermidine and spermine were increased in *Pseudomonas* infected lungs (p<0.001, respectively) but were unaffected by ABH treatment.

**Conclusions:**

Systemic arginase inhibition with ABH during *Pseudomonas* pneumonia in mice results in an increase in pulmonary NO formation but no pro-inflammatory effect.

## Introduction

Infection of the lung with bacteria leads to increased expression of the inducible nitric oxide synthase (iNOS or NOS2) and NO production [1]–[3], as does intra-tracheal instillation of lipopolysaccharide (LPS) [4], [5]. NO production from NOS depends on the availability of substrate and co-factors, as well as the presence of endogenous inhibitors including asymmetric dimethylarginine (ADMA) [6]. In the context of lung infection with *P. aeruginosa*, an opportunistic pathogen frequently causing infections in patients with chronic lung diseases including chronic obstructive pulmonary disease (COPD), bronchiectasis or cystic fibrosis (CF), evidence suggests that relative airway NO deficiency may have negative effects for the host. Studies in CF patients for instance have shown that low levels of airway NO are a risk factor for acquisition of this pathogen [7]. In addition, in a rat model of chronic *P. aeruginosa* lung infection, supplementation with L-arginine reduced the pro-inflammatory cytokine interleukin (IL)-1β in airways, inhibited neutrophil recruitment, and ameliorated lung tissue damage, while pharmacological inhibition of NOS in this model significantly worsened lung damage [3].

Arginase is an enzyme that converts L-arginine to urea and L-ornithine. The two isoforms of arginase are expressed in a number of tissues including the lung and are thought to reduce NO production from NOS by limiting the availability of substrate L-arginine [6], [8], [9]. Thus arginase may represent a target for interventions aiming to increase L-arginine availability for NOS and NO production. Inhibition of arginase in animal models of allergic airway inflammation, for instance, resulted in anti-inflammatory effects and abrogation of airway remodeling and hyperresponsiveness to methacholine in these animals, presumably by increasing L-arginine availability for NOS and increased NO formation [10]–[12]. Data on whether inhibition of arginase can increase NO production in the context of bacterial infection in-vivo are currently lacking. We therefore studied the effects of chronic systemic arginase inhibition on the pulmonary L-arginine metabolism in a mouse model of chronic *P. aeruginosa* lung infection.

## Methods

The experiments were approved by the institutional Animal Care Committee and were conducted in accordance with the guidelines of the Canadian Council for Animal Care.

### Mice and infection protocol

Eight to ten week old female C57BL/6 mice purchased from Charles River Laboratories (Charles River, Oakvile, Quebec, Canada) were housed in a pathogen-free environment and received autoclaved food and water in the laboratory animal services at our institution. Agarose beads embedded with *Pseudomonas aeruginosa* (mPAO1) were made following a published protocol [13] and modified by us, and beads were injected into the airways after intubation under direct vision as previously described [13] in anaesthetized mice (ketamine 150 mg/kg and xylazine 10 mg/kg administered intraperitoneally). A final *P. aeruginosa* dose of 2×10^6^ CFU in a volume of 40–50 µl was injected into the trachea. Infected mice were treated with a total of 4 i.p. injections of PBS or 100 µg of the arginase inhibitor 2(S)-amino-6-boronohexanoic acid (ABH) dissolved in 0.3 ml of PBS at 24, 48, 60 and 70 hours following the instillation of PAO-1. Body weight was monitored daily, before and for 3 days following the infection. At 72 hours post infection mice were anaesthetized, blood was drawn by intracardial puncture and organs were harvested. Uninfected not ABH treated mice were used as controls.

A different group of animals (male BALB/c mice, 8 weeks old) underwent an established LPS pneumonia protocol [14]. Anaesthetized mice were instilled with 50 µg of LPS from E. coli O111:B4 (Sigma) and treated with i.p. injections of PBS (n = 8) or ABH (n = 8) similar as above, immediately before, and 12, 24, 36 and 48 hrs post instillation of LPS. Lungs were harvest immediately after the last injection of ABH or LPS and processed on ice. Lysis buffer (25 mM Tris-HCl, pH 7.4, 1% TritonX100, 10% glycerol) containing protease inhibitors (Complete, Mini, EDTA-free plus 2 µM EDTA, Roche Applied Science) was added (2.5 ml/g lung). Tissue was homogenized using high-speed homogenizer (Polytron PT 1200E, Kinematica, Switzerland) for 1 min 3 times. Samples were then centrifuged (14000 rpm, 4°C) for 20 min, supernatants aliquoted and stored at −80°C until further analyses.

Liquid chromatography-tandem mass spectrometry (LC/MS/MS) to measure L-arginine, L-ornithine, L-citrulline, ADMA and the L-ornithine derived polyamines putrescine, spermidine and spermine was performed in supernatant of organ homogenates as previously described [15], [16]. NO metabolites in plasma and in lung homogenates from the LPS pneumonia model were quantified with help of a chemiluminescence analyzer (Eco Physics CLD 88 sp, Dürnten, Switzerland), while Griess reagent (Cayman, Ann Arbor, MI) was used for nitrate and nitrite measurements in lung homogenates of animals infected with *Pseudomonas*. Arginase activity was measured by conversion of L-arginine to ornithine in-vitro, as previously described [17]. A multi-analyte panel enzyme-linked immunosorbent assay (ELISA) was used to determine the concentrations of interferon-γ (IFN-γ), interleukin-1 beta (IL-1β), IL-6, macrophage inflammatory protein 2 (MIP-2), keratinocyte chemoattractant (KC), and tumor necrosis factor-alpha (TNF-α) in supernatant of lung homogenates (Millipore, Billerica, MA, USA).

All results are expressed as the mean ± standard error of the mean (SEM). Binary comparisons were made with two-tailed student's t-test or Mann-Whitney test, where appropriate. Comparisons of three groups were performed by one-way ANOVA with Turkey's multiple comparison or Kruskal-Wallis test with Dunn's multiple comparison post hoc test, where appropriate. P-values<0.05 were considered statistically significant. Statistical analyses were conducted using GraphPad Prism 4.0c (Graphpad Software Inc., La Jolla, CA USA).

## Results

*P. aeruginosa* lung infection resulted in significant weight loss but no mortality in animals. Weight loss following infection did not differ significantly (ANOVA) between ABH and PBS treated mice on day 1 (5.9±0.9 vs. 5.4±0.4%), day 2 (10.1±0.9 vs. 9.4±1.2%) or day 3 (7.5±1.7 vs. 6.7±1.9%). There was no difference in lung wet weights between ABH and PBS treated infected mice (0.167±0.017 g vs. 0.193±0.007 g, p = 0.244).

**Figure 1:**
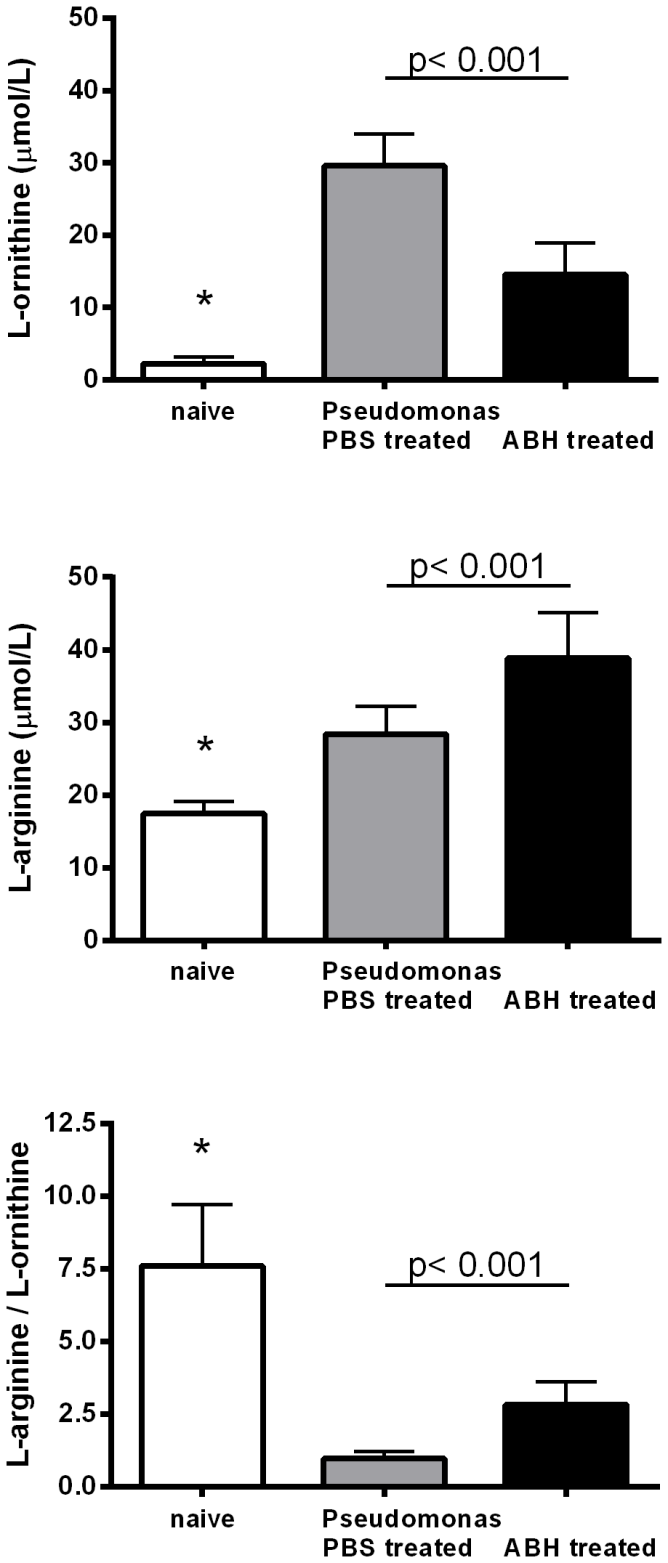
Concentrations of the amino acids L-ornithine and L-arginine in mouse lung homogenates of untreated (naïve) control as well as *Pseudomonas* infected mice treated with PBS or the arginase inhibitor 2(S)-amino-6-boronohexanoic acid (ABH). L-ornithine, the product of arginase activity, as well as L-arginine was significantly lower in naïve, compared to infected lungs (*: p<0.0001, ANOVA, respectively). ABH treatment resulted in a significant decrease in L-ornithine but increase in L-arginine concentration (p<0.001, t-test, respectively). The L-arginine/L-ornithine ratio, which can be used as an index of L-arginine availability for intracellular NOS, was reduced in infected lungs (*: p<0.0001, ANOVA) but improved in the ABH treatment group (p<0.001, t-test).

*Pseudomonas* infection resulted in a significant increase in L-arginine concentrations (28.4±1.4 vs 17.5±0.7 µM, p<0.0001) and a more pronounced increase in L-ornithine (29.6±1.7 vs 2.3±0.4 µM, p<0.0001) in lung homogenates of infected mice compared to non-infected controls. Consequently, the L-arginine/L-ornithine ratio, an index of L-arginine availability for intracellular NOS [18], [19], was significantly reduced by *P. aeruginosa* infection (Figure 1). Treatment with the arginase inhibitor ABH, resulted in a significant decrease in L-ornithine (14.6±1.6 µM, p<0.0001), the product of arginase activity, but increase in its precursor L-arginine (38.9±2.2 µM, p = 0.001). The L-arginine/L-ornithine ratio increased significantly with ABH treatment but did not normalize (Figure 1). L-citrulline concentrations were higher in the infected animals compared to non-infected controls (47.2±4.1 vs. 6.3±0.4 µM, p<0.0001) but not different in PBS vs ABH treated animals (49.5±9.2 µM).

**Figure 2:**
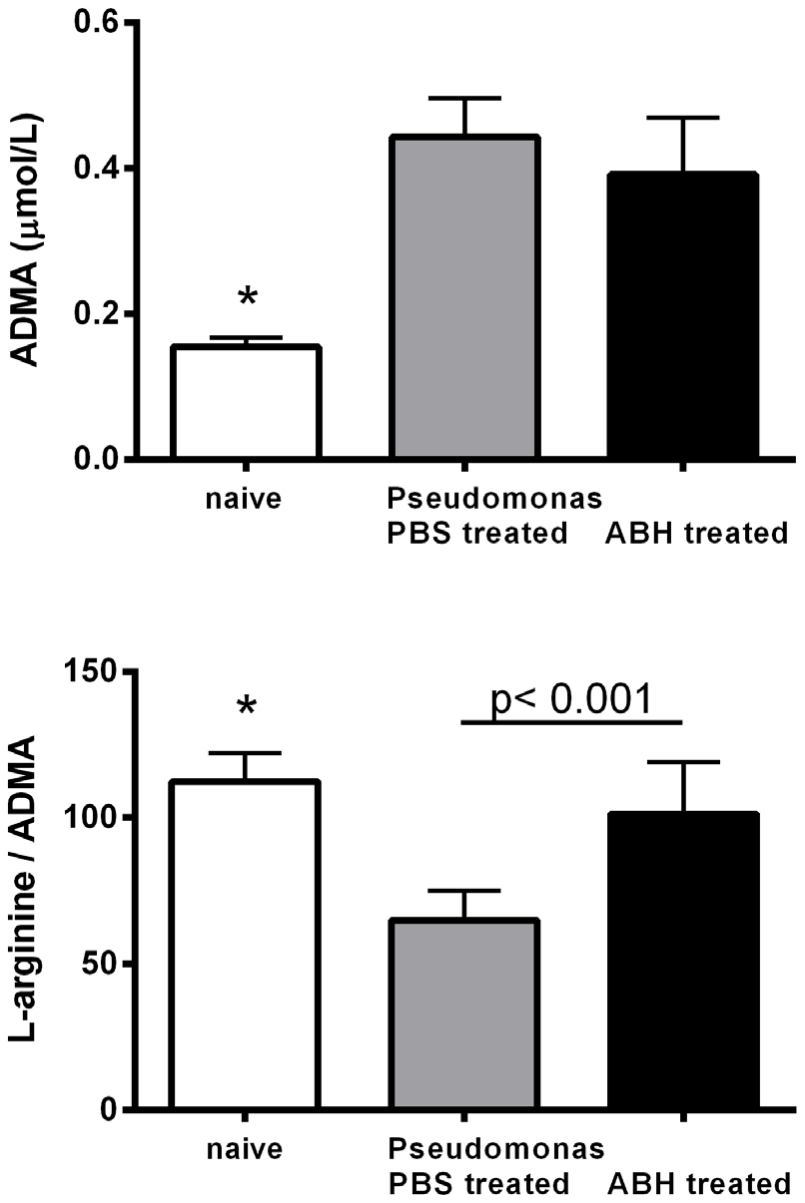
Concentrations of the nitric oxide synthase inhibitor asymmetric dimethylarginine (ADMA) in mouse lung homogenates of untreated (naïve) control as well as *Pseudomonas* infected mice treated with PBS or the arginase inhibitor 2(S)-amino-6-boronohexanoic acid (ABH). ADMA was significantly lower in naïve compared to *Pseudomonas* infected lungs (*: p = 0.0001, Kruskal-Wallis test). The L-arginine/ADMA ratio, which can be used as an index of NOS impairment, was significantly reduced in infected lungs (*: p<0.0001, ANOVA). Treatment with ABH did not change ADMA concentrations but resulted in normalization of the L-arginine/ADMA ratio. (p<0.001, t-test).

*P. aeruginosa* infection also resulted in a significant increase in the concentration of the competitive NOS inhibitor ADMA (0.44±0.02 vs. 0.16±0.01 µM, p<0.0001) and a decrease in the L-arginine/ADMA ratio, an index of NOS impairment. ABH treatment did not affect ADMA concentrations in the lung but restored the L-arginine/ADMA ratio to normal (Figure 2).

**Figure 3:**
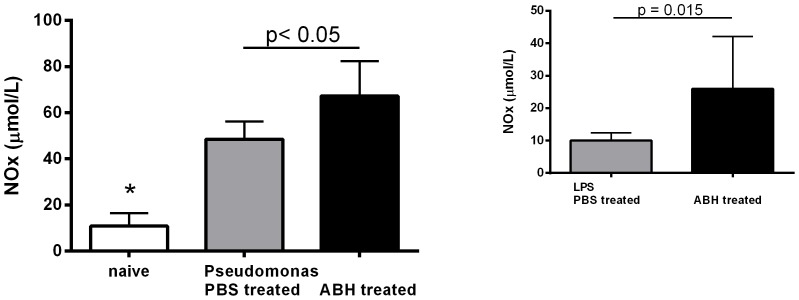
Nitric oxide metabolite (NOx) concentrations in mouse lung homogenates of untreated (naïve) control as well as *Pseudomonas* infected mice treated with PBS or the arginase inhibitor 2(S)-amino-6-boronohexanoic acid (ABH). Concentrations were significantly different between groups (p<0.0001, ANOVA). NOx levels in lung were lower in naïve compared to *Pseudomonas* infected and PBS treated mice (*: p<0.001, Mann-Whitney test). ABH treatment resulted in a significant increase in ABH vs. PBS treated animals (p<0.05, Mann-Whitney test). A similar increase in lung NOx after ABH treatment was seen in mice after intratracheal instillation of lipopolysaccharide (LPS) (p = 0.015, t-test).

Plasma NOx concentrations were higher in *Pseudomonas* infected PBS treated animals than controls (41.8±6.1 vs. 27.5±2.1 µM, p = 0.02) but not different from ABH treated animals (43.9±7.5 µM). NOx (nitrate+nitrite) concentrations were significantly increased in infected lungs compared to controls (48.5±2.9 vs. 10.9±2.3 µM, p<0.0001) and further increased with ABH treatment (67.3±5.7 µM, p = 0.01) (Figure 3). A significant increase in lung NO metabolite concentrations following arginase inhibition was also seen in the LPS pneumonia model (ABH vs. PBS: 26.0±5.7 vs. 10.1±0.8 µM, p = 0.01) (Figure 3).

NOx concentrations in liver homogenates were not different between the ABH or PBS treatment groups (23.1±3.2 vs. 24.1±5.2 µM) but ABH resulted in a significant increase in NOx in kidney homogenates (25.5±3.7 vs. 7.2±2.1 µM, p = 0.001). As arginase 1 is the predominant isoform expressed in liver while arginase 2 is the predominant arginase isoform expressed in kidney, the differences in NOx concentration between the two organs is suggestive of arginase 2 specificity of ABH.

Arginase activity measured in lung homogenates in-vitro confirmed an increase in infected mice (48.8±20.8 vs. 15±2.3 mU/mg protein, p<0.005). The expression of both arginase isoforms was increased in lung of infected mice using Western blot analysis (data not shown). Concentrations of the L-ornithine derived polyamines putrescine, spermidine and spermine were significantly higher in infected mice but not different between PBS and ABH treated animals (Table 1). Cytokine concentrations for the three groups are shown in Table 2. ABH treatment did not have an effect on cytokine concentrations.

**Table 1:** Concentrations (µmol/L) of the L-ornithine derived polyamines putrescine, spermidine and spermine in lung homogenates of naïve control and *Pseudomonas* infected mice treated with PBS or arginase inhibitor ABH.

	Putrescine	Spermidine	Spermine
**Control**	4.9±1.2 *	4.0±1.2 *	2.6±0.7 *
**Pseudomonas**			
**PBS**	34.2±2.4	39.6±2.3	13.6±0.8
**ABH**	28.7±2.9	37.5±3.7	12.5±0.8

* Concentrations were significantly different between groups (p<0.001, ANOVA). All three polyamines were lower in controls compared to infected animals but not different between PBS or ABH treatment group (n = 6–8/group).

**Table 2:** Cytokine concentrations (ng/g) in lung homogenates of naïve control and *Pseudomonas* infected mice treated with PBS or arginase inhibitor ABH.

		P. aeruginosa	
	Control (n = 6)	PBS (n = 14)	ABH (n = 15)
**IFN-γ**	0.04±0.01 *	3.50±0.83	2.54±0.52
**IL-1β**	8.54±0.96	15.51±3.02	12.26±2.37
**IL-6**	9.97±0.51 *	52.27±5.67	65.09±12.1
**MIP-2**	0.15±0.01 *	59.46±7.30	44.29±3.67
**KC**	4.52±1.43 *	31.84±5.90	29.28±3.58
**TNF-α**	0.05±0.01 *	7.61±1.41	5.48±0.83

Concentrations were significantly different between groups (p<0.001, Kruskal-Wallis test). All cytokines with the exception of IL-1β were lower in controls compared to infected animals not different between PBS and ABH treated animals.

## Discussion

We here show that *Pseudomonas* infection of the lung resulted in a significant increase in lung tissue L-ornithine, the product of arginase activity. While there was also an increase in L-arginine concentration in the lung following infection, the availability of L-arginine for NOS, expressed as L-arginine/ornithine ratio, was lower in infected mice than in non-infected controls. In addition, we observed an increase in the concentration of the competitive NOS inhibitor ADMA in infected lungs. Systemic application of an arginase inhibitor resulted in increased L-arginine availability for NOS and increased NO production. Increased NO production induced by arginase inhibition was not associated with an increase in pro-inflammatory cytokines in *P. aeruginosa* infected animals.

Arginase and NOS both compete for L-arginine as substrate and an important role of arginase is thought to be the regulation of NO production by limiting L-arginine availability for NOS [6], [8], [9]. While excessive NO production may have negative effects on inflammation, it is also possible that substrate limitation for NOS results in deleterious effects due to uncoupling of the enzyme resulting in superoxide radical formation [3], [20]–[22]. An example for the importance of the arginase/NOS balance for host/pathogen interactions is *Campylobacter pylori*, which induces arginase activity as a strategy to limit L-arginine availability for host NOS in order to reduce NO-mediated host defense and facilitate persistent gastric mucosal infection [23]. *Pseudomonas* is highly sensitive to NO- and nitrite-mediated killing, and therefore, limitation of NO production during infections with this bacterium may promote infection [24]. We have recently shown using stable isotopes, that infection of the mouse lung with *P. aeruginosa* resulted in a significant increase in the expression and activity of NOS 2 but also arginase 1 and arginase 2 [25]. In the current study we demonstrated that systemic arginase inhibition enhances pulmonary NO production using an established model of *P. aeruginosa* lung infection in the mouse. The observed changes in NO production in the infected and ABH treated animals were not caused by effects of the arginase inhibitor on *Pseudomonas*, as similar increases in NO following the administration of ABH were also seen in animals with intratracheal LPS instillation. Gender disparity exists in certain aspects of the nitric oxide pathway [26]–[29], and it is therefore worth mentioning that the infected animals in our experiments were females while the LPS treated were male mice. As arginase inhibition resulted in increases in NO production in both groups, it can be speculated that the effect of ABH on pulmonary NO production in the mouse is gender independent. However, further studies will be needed to assess whether treatment with arginase inhibitors may result in different physiological responses in males and females.

Our results also showed that infection of the lung resulted in a significant increase in the concentration of the competitive NOS inhibitor ADMA and a decrease in L-arginine/ADMA ratio (NOS substrate/inhibitor) an index reflecting NOS impairment. Effects of ADMA in the context of respiratory infections with *Pseudomonas* were previously studied using human nasal epithelial cells. Pre-incubation with ADMA significantly reduced *Pseudomonas*-induced epithelial damage, loss of ciliated cells and bacterial adherence to the cultured respiratory mucosa in-vitro [30]. While these data provided evidence that ADMA was associated with beneficial effects, it was unclear in that study whether the observed effects of ADMA were direct or mediated through inhibition of NOS, as ADMA did not cause a significant change in nitrite concentration in the culture medium [30]. In our experiments ABH treatment did not result in a change in ADMA concentration in the infected lungs, but normalized the ratio of L-arginine/ADMA with a concomitant increase in NO production.

Dosing of the arginase inhibitor was based on previous publications on systemic use of ABH in rodents [31], [32]. The half-life of ABH in C57BL/6 mice was previously reported to be approximately 8 hours [33]. There was no evidence that repeated systemic application of the arginase inhibitor was harmful to the animals. Although *Pseudomonas* resulted in significant weight loss in the animals on day 1 and day 2 of the infection, there were no differences in weight loss between the groups of ABH or PBS treated animals. There was also no significant difference in cytokine concentrations in lung homogenates when comparing ABH and PBS treated mice, suggesting that neither ABH nor the increase in NO production in the lung had a pro-inflammatory effect. Previous work has shown that mice deficient for NOS2 had impaired clearance of *Pseudomonas* from the lung 18 h after infection [34]. The present study focused on the effect of arginase inhibition on NO production; whether the increase in NO formation would lead to altered defense against *Pseudomonas* was not assessed, but should be investigated in future studies.

L-ornithine, the product of arginase activity, is substrate for collagen formation but also for polyamine biosynthesis. Polyamines are important in cell repair and also act as NOS inhibitors [6], [9], [16]. Arginase activity and polyamine levels are significantly increased in models of asthma and in the guinea pig for instance, the increase in putrscine was prevented by pharmacological blockade of arginase [11]. In our experiments, *Pseudomonas* infection also resulted in a significant increase in polyamine concentrations in the lung. Arginase inhibition reduced L-ornithine formation by approximately 50% but there was no effect on polyamine concentrations in the lung. This could likely be due to the fact that ornithine availability for ornithine decarboxylase (ODC), the first and rate-limiting step in L-ornithine dependent polyamine biosynthesis [35] remained sufficient for polyamine production.

An alternative strategy to increase concentration and availability of L-arginine for NOS is supplementation of its substrate. A previous study using a chronic model of *Pseudomonas* infection of the lung in rats had shown that L-arginine given in drinking water resulted in lower IL-1β concentrations in BAL fluid in the L-arginine treated compared to the untreated group, whereas VEGF was increased. L-arginine supplementation has the potential disadvantage of providing substrate for NOS but also arginase enzymatic activity, which may result in unwanted effects of L-ornithine derived metabolites. For instance, generation of spermine by ODC inhibits iNOS translation and NO-mediated H. pylori killing [36], [37]. Studies in humans with CF or asthma, both conditions associated with increased arginase activity and relative NO deficiency, have shown that the effect of systemic L-arginine supplementation on pulmonary NO formation is moderate and limited by increased formation of the NOS inhibitor ADMA, a product of protein degradation [38], [39]. Ultimately, one approach does not exclude the other and the combination of both may have the highest likelihood of addressing the relative NO deficiency in *P. aeruginosa* infection.

In conclusion systemic ABH used in the early phase of acute *P. aeruginosa* lung infection at doses effective to significantly reduce arginase activity in the lung result in increased pulmonary NO production. The role of pharmacological inhibition of arginase for treatment of lung infections deserves further investigation.
