# Correction: Effect of Arginase Inhibition on Pulmonary L-Arginine Metabolism in Murine *Pseudomonas* Pneumonia

**DOI:** 10.1371/journal.pone.0101923

**Published:** 2014-06-26

**Authors:** The PLOS ONE Staff

The third author’s name is incorrect. The correct name is David Nobuhiro Douda. The correct citation is: Mehl A, Ghorbani P, Douda DN, Huang H, Palaniyar N, et al. (2014) Effect of Arginase Inhibition on Pulmonary L-Arginine Metabolism in Murine *Pseudomonas* Pneumonia. PLoS ONE 9(3): e90232. doi:10.1371/journal.pone.0090232
